# Combination of Pulse Steroid with Intratympanic Injections in Sudden Sensorineural Hearing Loss

**DOI:** 10.22038/ijorl.2020.43887.2452

**Published:** 2021-01

**Authors:** Maryam Amizadeh, Keramat Mozafarnia, Javad Moslemikia, Ahmad Naghibzadeh-Tahami

**Affiliations:** 1 *Clinical Research Development Unit, Shafa Hospital, Kerman University of Medical Sciences, Kerman, Iran.*; 2 *Clinical Research Unit, Jiroft University of Medical Science, Jiroft, Iran.*; 3 *Modeling of Health Research Center, Institute for Futures Studies in Health, Kerman university ofMedical Science, Kerman, Iran.*

**Keywords:** Intratympanic, Pulse therapy, Sudden sensorineural hearing loss

## Abstract

**Introduction:**

Oral prednisolone was suggested as the first step to treat idiopathic sudden sensorineural hearing loss (ISSHL). This study aimed to investigate the effect of pulse therapy with methylprednisolone and intratympanic methylprednisolone, compared to traditional oral prednisolone therapy on patients with ISSHL.

**Material and Methods:**

This randomized control trial included an experimental group receiving 500 mg intravenous methylprednisolone for three sequential days, followed by 1 mg/kg oral prednisolone for 11 days, and intratympanic Depo-Medrol four times twice a week. On the other hand, the control group received 1 mg/kg oral prednisolone for 14 days. Hearing change was assessed through pure tone audiometry. Subsequently, hearing recovery was investigated and analyzed in this study.

**Result:**

This study was conducted on 51 patients who were divided into two groups of experimental (n=26) and control (n=25). The result revealed no significant difference between the two groups in terms of hearing improvement (P=0.28).

**Conclusion:**

This revealed no added benefit in pulse steroids combined with intratympanic injections in cases with sudden hearing loss.

## Introduction

Idiopathic sudden sensorineural hearing loss (ISSHL) is designated as 30 dB sensorineural hearing loss or more over at least three frequencies during three days or fewer (1). It has an incidence of 5-10 per 100000 papulation in a year (2). Similarly, viral infections, cellular stress theory, circulatory disorder, membrane damage of labyrinth, and autoimmune reactions have been suggested for the pathogenesis of this disorder (3). Oral steroid is recommended for ISSHL, and it is noteworthy that about 50% of the patients cannot be treated through this medicine. According to guidelines, the intratympanic steroid has been recommended when oral therapy is not beneficial for patients suffering from ISSHL (4).

Moreover, some studies use intratympanic as primary treatment (5,6). In the same vein, pulse steroid therapy has been recommended for the nephritic syndrome, systemic lupus erythematous, optic neuritis, rheumatoid arthritis, and other autoimmune diseases (7). 

It should be noted that glucocorticoids have anti-inflammatory and immune-suppressive properties (8). Regarding the possible role of autoimmunity in this disorder, this clinical trial aimed to conclude that pulse and intratympanic therapies would be administered together as the primary treatment without delay. 

## Materials and Methods

This prospective double-blind clinical trial was carried out from June 2012 to September 2019 in a referral university hospital. In total, 90 patients (age range:11-60 years) with ISSNHL disorder were included in this study and underwent three para clinic tests, namely pure tone audiometry, contrast-enhanced magnetic resonance imaging, and lab workshop. Table 1 tabulates the inclusion and exclusion criteria. out of 90 patients, 29 cases were excluded from the study, and the remaining 61 individuals were randomly assigned into two groups of control (n=28) and experimental (n=29). 

**Fig 1:**
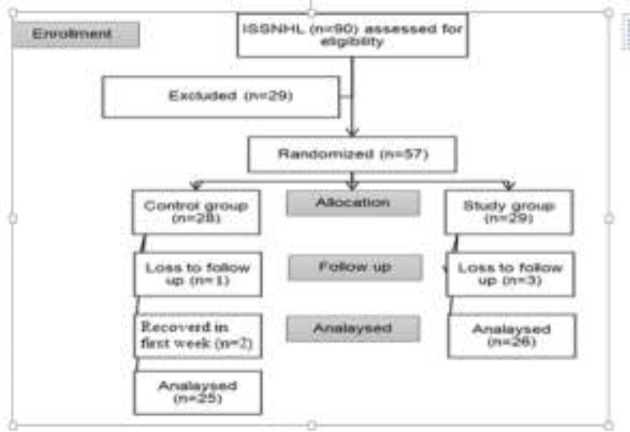
Study of the flow diagram (ISSNH= Idiopathic Study Sudden Sensorineural Hearing loss)

Totally, four patients were lost to follow up, and two cases in the experimental group were recovered in the 5^th^ and 7^th^ weeks during treatment (Fig.1). Eventually, 26 and 25 patients in the experimental and control groups completed this protocol, respectively.

**Table 1:** Inclusion and Exclusion Criteria

**Inclusion Criteria**
1. Sensorineural hearing loss of 30 dB or more covering at least three contiguous audiometric frequencies, which occur within three days or fewer2. No identifiable cause despite an adequate investigation3. Normal or near-normal hearing in the contralateral ear4. Age range: 11-60 years5. No history of previous treatment6. No contraindication for proposed therapy
Exclusion Criteria
1. Any identified etiology during therapy2. Previous disease or surgery in the affected ear 3. Pregnant or lactating females 4. Uncontrolled diabetes or autoantibody diseases

The study protocol was approved by the Clinical Research Ethics Committee of Kerman University of Medical Sciences, Kerman, Iran (Ir.Kmu.ah.rec.1397.001).

**Protocol treatment**

The experimental group received 500 mg intravenous methylprednisolone for three sequential days (9,10). It was then followed by oral prednisolone 1 mg/kg (maximum 60 mg). In addition, intratympanic injection 0.3-0.6 cc of Depo-Medrol (40mg/ml) was administered four times every other day since the first day of the treatment. On the other hand, the control group received 1 mg/kg oral prednisolone (maximum 60mg) for 14 days. 

All patients underwent hearing evaluation using pure tone audiometry after the end of treatment and three months later. Meanwhile, hearing recovery classification was performed according to the guidelines of the American Academy of Otolaryngology-Head and Neck Surgery Foundation (AAO-HNSF) (Table.2).

**Table 2:** Hearing Recovery Classification According to the American Academy of Otolaryngology-Head and Neck surgery

1. Complete recovery: Return to within 10 dB HL of the unaffected ear and recovery of word recognition scores to within 5% to 10% of the unaffected ear.2. Partial recovery: Should be defined in 2 ways based on whether or not the degree of initial hearing loss after the event of SSNHL rendered the ear nonservice able (based on the AAO-HNSF definition).a: For ears that were rendered nonservice able by the episode of SSNHL, return to serviceable hearing should be considered a significant improvement (partial recovery) and recovery to less than serviceable levels as “no recovery”.b: For ears with SSNHL to hearing levels that are still in the serviceable range, a 10-dB HL improvement in pure-tone thresholds or an improvement in WRS of > 10% should be considered partial recovery.3. No recovery: Anything less than a 10-dB HL improvement should be classified as no recovery.

Nonservice able hearing: <50% Speech discrimination score and > 50 dB on pure tone average.AAO-HNSF=American Academy of Otolaryngology-Head and Neck Surgery Foundation; HL=hearing loss; SSNHL=sudden sensorineural hearing loss; WRS=word recognition scores.

## Results

***This study included 51 patients who were assigned into two groups of experimental (n=26) and control (n=25).*** Table 3 ***summarizes the demographics and baseline audiology characteristics of the patients at the beginning of the study. There was no significant statistical difference between the two groups (P≥0.05).***

Table 4 presents a comparison of hearing improvement between the two groups three months after treatment. Accordingly, there was no significant statistical difference between the two groups in this regard (P=0.3).

**Fig 2:**
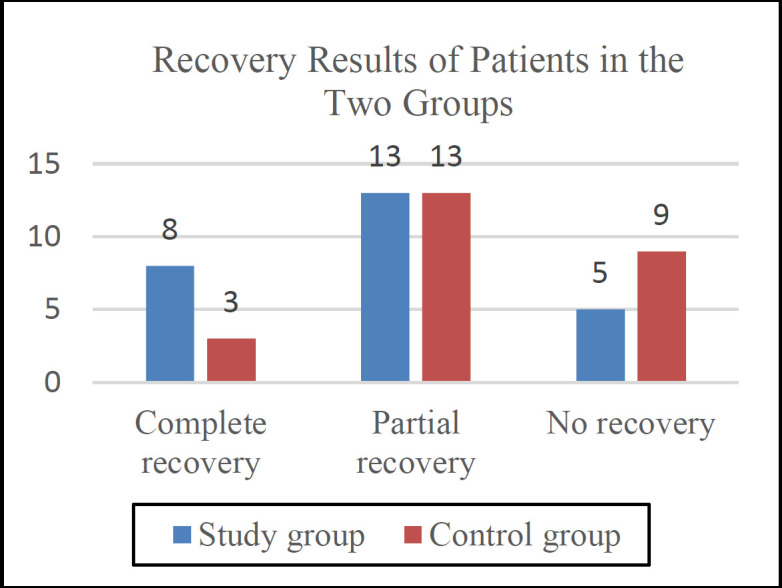
Recovery results of patients in the two groups

As can be seen in Figure 2, there is no significant difference between the two groups regarding hearing recovery.

**Table 3:** Demographics and baseline audiology characteristics of the patients in the two groups

	Experimental group (n=26)	Control group(n=25)	**P-value**
Gender-male: Gender-female (n)	15:11	14:11	**0.903**
Vertigo	6	4	**0.52**
Days from onset to treatment	6.42±3.74	5.44±3.30	**0.32**
**Severity of hearing loss (n)**			
Mild	2	4	**0.82**
Moderate	5	3	
Moderately-sever	4	4	
Sever	2	3	
Profound	13	11	
Tinnitus	18	18	
**Hearing level in each frequency (dB)**			
0.25 KHz	72.5±32.00	67.6±33.48	**0.59**
0.5 KHz	74.80±30.08	70.2±33.30	**0.60**
1 KHz	77.69±25.54	73.6±29.91	**0.60**
2 KHz	76.73±27.92	73.8±29.7	**0.71**
3 KHz	77.69±28.00	75±27.87	**0.73**
4 KHz	76.34±32.42	76.4±26.63	**0.99**
PTA (dB)	76.57±26.77	72.46±30.0	**0.60**
WRS (%)	76.75±20.07	86.69±8.40	**0.11**

**Table 4:** Hearing improvement three months after treatment in the two groups

Hearing Improvement	Experimental group (n=26)		Control group (n=25)		P-value
Hearing improvement at each frequency (dB)	Mean	SD	Mean	SD	
**0.25 KHz**	44.80	26.77	45.8	31.94	0.90
**0.5 KHz**	45.76	26.78	50.6	6.41	0.56
**1 KHz**	44.23	25.91	52.4	32.11	0.32
**2 KHz**	41.15	26.84	52.8	30.03	0.15
**3 KHz**	44.03	28.42	48.82	30.15	0.25
**4 KHz**	48.26	28.52	56.2	31.79	0.35
**PTA improvement (dB)**	43.53	26.07	47.74	28.29	0.28
**WRS improvement (%)**	60.5	17.53	5.66	7.94	0.30

## Discussion

This study is the first attempt to investigate the effect of pulse steroid combination with intratympanic steroid injection simultaneously in patients suffering from ISSHL disorder. Treatment of ISSNHL is one of the most challenging issues in otolaryngology. Some modalities, such as antivirus, vasodilators, and hyperbaric oxygen were suggested in this regard; however, steroid therapy is the most acceptable therapeutic approach (11). Furthermore, the most accepted protocol is oral glucocorticoid; nonetheless, the intratympanic steroid can be considered a salvage treatment on the failure of systemic steroids (12-14). According to the results of other studies, the effect of the intratympanic steroid includes ion hemostasis, apoptosis inhibition, antioxidant effect, and the importance of local microvascular flow (15,16). 

Additionally, systemic steroids can result in the reduction of immune system activity and decrease the number of circulatory leucocytes (17). Pulse therapy would be related to the infusion of high-dose glucocorticoids in a short burst (9). Methylprednisolone is an intermediate-acting and anti-inflammatory agent with a low inclination to induce sodium and water retention, compared to hydrocortisone with a dose of 20-30mg/kg per pulse for three days (9-10). It was supposed that all the therapeutic mechanisms of this medication were utilized for the treatment of this disease; however, the results were quite different. Narozny et al. (18) and Westerlaken employed pulse steroid in their studies. According to a study conducted by Narozny (19), the patients who received pulse steroid (1000 mg methylprednisolone for 3 days) made a significant recovery. To our knowledge, this result might be due to consuming hyperbaric oxygen simultaneously. In addition, the results of a study performed by Westerlaken showed no significant difference that can be attributed to receiving no suitable dosage of steroid by the control group.

The experimental group in a study carried out by Efttekharian (2015) (20) received pulse steroid, whereas this group received both pulse steroid and intratympanic steroid injection in the present study. It means that all the steroid mechanisms (local and systemic effect) were utilized in this study. The result showed that the utilization of the steroid mechanisms might not be appropriate due to the insufficient volume of the sample. Jang Bin Lee (2015) mentioned that the combined therapy (systemic prednisolone and intratympanic injection of dexamethasone) (21) had a better effect on hearing recovery, compared to systemic prednisolone and the results of a study conducted by Arastou et al. (22). Additionally, Dajang (2016) infused dexamethasone into patients for 10 days. The result was consistent with the findings of the above-mentioned studies. 

Although Dajang found a favorable result in his study, the patients were hospitalized for 10 days, which could be time taking and somehow costly (23).

Considering the results of a study conducted by Bae (2013), the experimental group who received combination therapy (systemic and intratympanic steroid) showed no significant statistical differences (24). It is noteworthy that the large sample size was advantageous in the study conducted by Boa. 

Regarding the limitation of this study, one can name the sample size. The differences in the studies can be attributed to the sample size, scale of hearing recovery, selective sort of injected medication, time of consuming medicine, and dosage. The utilization of supplemental medications can lead to obtaining different results in the above-mentioned studies. Intratympanic injection of Depo-Medrol was performed in a study conducted by Arslans (25). 

The same medication was also utilized in the current study. On the other hand, Battaglia believed that tissue-binding affinity in methylprednisolone was not as much as that in dexamethasone (26). Future studies are recommended to consider a larger sample size and utilize different medications and dosages to obtain improvements in hearing recovery. 

## Conclusion

According to the results, combination therapy with pulse steroid along with intratympanic injection steroid had a comparative effect on the improvement of hearing loss in these patients.

## References

[1] Haberkamp TJ, Tanyer HM (2007). Management of idiopathic sudden sensorineural hearing loss. AmOtol.

[2] Byl FM (2011). sudden hearing loss: eight years’ experience and suggested prognostic table. Laryngoscope.

[3] Stokroos RJ, Albers FW, Tenverget EM (1998). Antiviral treatment of idiopathic sudden sensorineural hearing loss. Acta otolaryngal.

[4] Stachler RJ, Chandrasekhar SS (2012 ). Clinical practice guideline: sudden hearing loss. Amrican Academy of otolaryngology –head and neck surgery.

[5] Dermian H (2018). Contribution of interatympanic steroids in the primary treatment of sudden hearing loss. ACATA OTO-LARYNGOLOGICA.

[6] Rauch SD, Halpin CF, Antonelli PJ, Babu S, Carey JP, Gantz BJ (2011). Oral vs interatympanic corticosteroid therapy for idiopathic sudden sensorineural hearing loss: randomized trial. JAMA.

[7] Sinha A, Begga A (2008). Pulse steroid therapy. Indian J Pediatr.

[8] Woods JE, Anderson CF, Doweerd JH (1973). High dosage intravenously administered methylprednisolone in renal transplantation A preliminary report. Jama.

[9] Panat SR, Aggarwal A, Joshi A (2012). Pulse therapy: A boon or bane. J Dent Sci Oral Rehabil.

[10] Gupta G, Jain A, Narayanasetty NK (2014). Steroid pulse therapies in dermatology. Muller J Med Sci Res.

[11] Awad Z, Hunis C, Pothier DD (2002). Antiviral for idiopathic sudden sensorineural hearing loss. Cochrance Database syst rev.

[12] Garavello W, Gallzzi F, Gaini RM, Zanetti D (2010). Interatympanic panic steroid treatment for sudden deafness: a meta-analysis of randomized controlled trails. Otol neurotol.

[13] Crane RA, Camilon M, Nguyen S, Meyer TA (2017). Steroid for treatment of sudden sensorineural hearing loss: a meta-analysis of randomized controlled trails. Laryngoscope.

[14] Li H, Feng G, Wang H, Feng Y (2011). Intratympanic steroid therapy as a salvage treatment for sudden sensorineural hearing loss after failure of conventional therapy: a meta-analysis of randomized controlled trails. Clin ther.

[15] Kopke RD, Hoffer ME, Wester MD, Jakson RL (2001). Targeted topical steroid therapy in sudden sensorineural hearing loss. Otol neural.

[16] Stahn C, Lowenberg M, Hommes, DW, Buttgereiy F (2007). Molecular mechanism of glucocorticoid receptor agonists. Mol cell Endocinal.

[17] Ryan AF, Pak K, Low W (2012). Immunological damage to the inner ear: current and future therapeutic strategies. Adv Otorhinolaryngol.

[18] Naronzny W, Sicko Z, Kot J, Kucszkoski J (2004). Usefulness of high doses of glucocorticoids and hyperbaric oxygen therapy sudden sensorineural hearing loss treatment. Otol neurotol.

[19] Westerlaken BO, DE kleine E, Van der laan B, Albers F (2007). The treatment of idiopathic sudden sensorineural hearing loss using pulse therapy. Laryngoscope.

[20] Eftekharian A, Amizadeh M (2015). Pulse steroid therapy in idiopathic sudden sensorineural Hearing loss: A randomized controlled clinical trial. laryngoscope.

[21] Jong Bin Lee, Seong Jun Choi (2015). Potential Benefits of Combination Therapy as Primary Treatment for Sudden Sensorineural Hearing Loss. Otolaryngology-Head and Neck Surgery.

[22] Arastou S, Tajeddini A (2013). Borghei Combined intratympanic and systemic steroid therapy for poo- prognosis sudden sensorineural hearing loss. Iran J Otorhinolaryngeal.

[23] Da Jung Jung, Ji Hye Park (2016 ). The Efficacy of Combination Therapy for Idiopathic Sudden Sensorineural Hearing Loss. Laryngoscope.

[24] Bae SC, Noh HL, Jun BC (2013). Efficacy of intratympanic steroid therapy for idiopathic sudden sensorineural hearing loss: comparison with systemic steroid therapy and combined therapy. Acta Otolaryngol.

[25] Arslan N, Oguz H, Demirei M (2011). Combination intratympanic and systemic use of steroid for idiopathic sudden sensorineural hearing loss. Otol Neurotic.

[26] Battaglia A, Burchett R, Cueva R (2008). Combination therapy 9 intratympanic dexamethasone+high-dose prednisolone taper) for the treatment of idiopathic sudden sensorineural hearing loss. Otol Neurotol.

